# Supplementary material for: Controllable branching of robust response patterns in nonlinear mechanical resonators
Source: Nat Commun. 2023 Jan 11;14:161. doi: 10.1038/s41467-022-35685-5 (PMC9834403; doi:10.1038/s41467-022-35685-5)
Supplement: Supplementary file 1 — Supplementary Information [file 41467_2022_35685_MOESM1_ESM.pdf]

Supplementary information: Controllable branching of robust response patterns in nonlinear mechanical resonators

Axel M. Eriksson, Oriel Shoshani, Daniel López,
Steven W. Shaw, and David A. Czaplewski

November 28, 2022

Supplementary Section 1 Theory

To model the mechanical device that exhibits branching of robust response patterns (RRP), we use a minimalistic model (a so-called normal form [1]) that captures the qualitative behavior. The two coupled mechanical modes and their interaction are described by a pair of linearly-damped oscillators with modal coordinates q_i that are coupled via a single term potential $U_{\rm cpl} = g q_1^3 q_2$ with coupling strength g, given by

$$\ddot{q}_1 + \gamma_1 \dot{q}_1 + \omega_1^2 q_1 + \alpha_1 q_1^3 + 3 g q_1^2 q_2 = f \cos(\Omega t) \tag{S1}$$

$$\ddot{q}_2 + \gamma_2 \dot{q}_2 + \omega_2^2 q_2 + g q_1^3 = 0. \tag{S2}$$

The first mode has angular frequency ω_1, damping γ_1, and Duffing nonlinearity α_1, and is directly driven with a single tone harmonic of amplitude f and frequency Ω. The second mode amplitude remains sufficiently small such that it does not enter its nonlinear regime; hence, we model it as a linear mode with angular frequency ω_2 and damping γ_2.

Supplementary Section 1.1 Rotating wave approximation

The first mode is resonantly driven with $|\Omega - \omega_1|/\Omega \ll 1$ and it is close to a 1:3 internal resonance with the second mode, $|\omega_2/3 - \omega_1|/\Omega \ll 1$. The low dissipation of the mechanical modes (quality factors exceeding 5×10^4) allows us to apply the rotating wave approximation (RWA) by introducing complex amplitudes a_j, which are varying slowly on the timescale of Ω^{-1},

$$q_j(t) = a_j(t) e^{i\Omega_j t} + a_j^*(t) e^{-i\Omega_j t}, \tag{S3}$$

$$\dot{q}_j(t) = i\Omega_j (a_j(t) e^{i\Omega_j t} - a_j^*(t) e^{-i\Omega_j t}), \tag{S4}$$

where $\Omega_1 = \Omega$ and $\Omega_2 = 3\Omega$. The corresponding dynamical equations of the complex amplitudes $a_j(t)$ in the RWA, where the fast-oscillatory non-resonant terms have been disregarded, are approximated by

$$\dot{a}_1 = -i\left(\Delta_1 a_1 - \frac{3}{2\Omega}\alpha_1 a_1|a_1|^2 + \frac{3}{2\Omega} g a_1^* a_1^* a_2\right) - \frac{\gamma_1}{2}a_1 + i\frac{f}{4\Omega}, \tag{S5}$$

$$\dot{a}_2 = -i\left(\Delta_{IR} a_2 - \frac{1}{2\Omega}\alpha_2 a_2|a_2|^2 + \frac{g}{6\Omega}a_1^3\right) - \frac{\gamma_2}{2}a_2, \tag{S6}$$

where we have introduced the detuning from the natural frequency of the first mode $\Delta_1 = \Omega - \omega_1$ and detuning from the internal resonance $\Delta_{IR} = 3\Omega - \omega_2$. The complex amplitudes $a_{1,2} = |a_{1,2}|e^{i\phi_{1,2}}$ describe the amplitudes ($|a_{1,2}|/2$) and phases ($\phi_{1,2}$) of the periodic oscillation, which rotate with frequencies Ω and 3Ω, respectively.

Supplementary Section 1.2 Separation of timescales - fast/slow dynamics

We note that in our system (S5)-(S6), for the range of drive parameters of interest, the detuning of the first mode is considerably larger than the detuning of the internal resonance and the dissipation rates, i.e., $\Delta_1 \gg \Delta_{IR} \sim \gamma_1 \sim \gamma_2$. Therefore, the first mode will exhibit fast dynamics whereas the dynamics of the second mode is relatively slow. To better see this, we rescale the time as $\tau = t\Delta_1$ and the mode complex amplitudes as $a_{1rs} = a_1 3^{-1/4}\sqrt{\alpha_1/(2\Delta_1\Omega)}$, $a_{2rs} = a_2 3^{1/4}\sqrt{\alpha_1/(2\Delta_1\Omega)}$, and obtain the following set of equations for the mode quadratures ($X_{1,2} = |a_{1,2rs}|\cos\phi_{1,2}$, $Y_{1,2} = |a_{1,2rs}|\sin\phi_{1,2}$)

$$\dot{X}_1 = Y_1 - \sqrt{3}Y_1(X_1^2 + Y_1^2) - \epsilon\frac{\gamma_{1rs}}{2}X_1 - 3\epsilon g_{rs}[Y_2(X_1^2 - Y_1^2) - 2X_2X_1Y_1], \tag{S7}$$

$$\dot{Y}_1 = -X_1 + \sqrt{3}X_1(X_1^2 + Y_1^2) - \epsilon\frac{\gamma_{1rs}}{2}Y_1 + 3\epsilon g_{rs}[X_2(X_1^2 - Y_1^2) + 2Y_2X_1Y_1] - \epsilon f_{rs}, \tag{S8}$$

$$\dot{X}_2 = \epsilon\left(\Delta_{IRrs}Y_2 - \frac{\gamma_{2rs}}{2}X_2 - g_{rs}Y_1(3X_1^2 - Y_1^2)\right), \tag{S9}$$

$$\dot{Y}_2 = \epsilon\left(-\Delta_{IRrs}X_2 - \frac{\gamma_{2rs}}{2}Y_2 + g_{rs}X_1(X_1^2 - 3Y_1^2)\right), \tag{S10}$$

where $\epsilon g_{rs} = g/\alpha_1$, $\epsilon f_{rs} = 3^{-1/4} f\sqrt{\alpha_1/(32\Delta_1^3\Omega^3)}$, $\epsilon\gamma_{1,2rs} = \gamma_{1,2}/\Delta_1$, $\epsilon\Delta_{IRrs} = \Delta_{IR}/\Delta_1$, and the introduced small parameter, $\epsilon = 0.01$, resulting in $\gamma_{1rs} = 0.0438$, $\gamma_{2rs} = 0.109$, $g_{rs} = 1.20$ and drive parameters are set to $f_{rs} = 0.20$ and $\Delta_{IRrs} = 0.0803$, which correspond to the experimental parameters used to measure (Fig. 1e) in the main paper.

The separation of timescales [2, 3] is an important feature of the system which occurs due to the small parameter ϵ, since to a first approximation, the time derivatives of (X_1, Y_1) are of order 1, but the time derivatives of (X_2, Y_2) are on the much slower timescale ϵ. Note that this separation of timescales holds as long as

1. the expression within the parentheses of Eqs. (S9)-(S10) are of order 1 or smaller. As seen in Supplementary Fig. S1, this requirement is fulfilled.
2. the right-hand sides of Eqs. (S7)-(S8) are not close to zero, e.g., the system is not near a stationary point (in the fast variable subsystem). We will return to discuss when this condition breaks down.

When these conditions hold, on the fast timescale, the (X_1, Y_1) variables will execute fast oscillations according to Eqs. (S7)-(S8) where (X_2, Y_2) can be treated as constants ($\dot{X}_2 = \dot{Y}_2 = 0$). On the slow timescale, the period T of these oscillations slowly change with time and the slow dynamics of the (X_2, Y_2) variables can be approximated by

$$\dot{X}_2 \approx \epsilon \left(\Delta_{IRrs} Y_2 - \frac{\gamma_{2rs}}{2} X_2 - \frac{1}{T} \int_0^T g_{rs} Y_1 (3X_1^2 - Y_1^2)\ \mathrm{d}t \right), \tag{S11}$$

$$\dot{Y}_2 \approx \epsilon \left(-\Delta_{IRrs} X_2 - \frac{\gamma_{2rs}}{2} Y_2 + \frac{1}{T} \int_0^T g_{rs} X_1 (X_1^2 - 3Y_1^2)\ \mathrm{d}t \right), \tag{S12}$$

which describes damped oscillations that are driven by the average position of the fast variable oscillations. As seen in Supplementary Fig. S1, the slow variables (X_2, Y_2) do (of course) contain dynamics on the fast timescale, (the very small oscillations around the main trajectory). It is these dynamics that are disregarded in the approximation done in Eqs. (S11)-(S12). The huge benefit of disregarding these fine details is that we can treat (X_2, Y_2) as bifurcation parameters in the fast variable subsystem, Eqs. (S7))-(S8). The evolution of these bifurcation parameters are given by Eqs. (S11)-(S12). The torsional mode when viewed in the rotating plane is effectively an overdamped oscillator $\Delta_{IRrs} \sim< \gamma_{2rs}$ [see Eqs. (S11)-(S12)] driven by high frequency oscillations $\sim |\Omega - \omega_1|$. Hence, the torsional mode drifts towards the steady state dictated by the average value of the flexural mode, which sets a rough timescale of the slow dynamics to $\gamma_2 >\sim \gamma_1$.

Supplementary Section 1.3 Bifurcation analysis

After separating the timescales, standard bifurcation theory of the first mode can be carried out, where the system parameters $f_{rs} = 0.20$ and $\Delta_{rs} = 0.0803$ are kept constant whereas the second mode variables X_2 and Y_2 are treated as the bifurcation parameters in Eqs. (S7)-(S8). The only[1] bifurcations found are saddle-node (SN) bifurcations, which change the numbers of fixed points in the fast variable plane.

In bursting systems, the trajectory in the fast variable plane transitions between stable stationary (fixed point) and oscillatory (limit cycle) states [3]. The saddle-node bifurcations found in the numerical bifurcation analysis are therefore good candidates for being

[1]Note, details about the bifurcation diagram of the full system, Eqs. (S7)-(S10), which is presented in (Fig. 1c) of the main article can be found in [4]. The SNIC bifurcation [5], which is responsible for the transition between excitable to repetitive execution of RRPs, lives in the (X_1, Y_1, X_2, Y_2) phase space and is not related to the fast/slow dynamics discussed in this supplementary information.

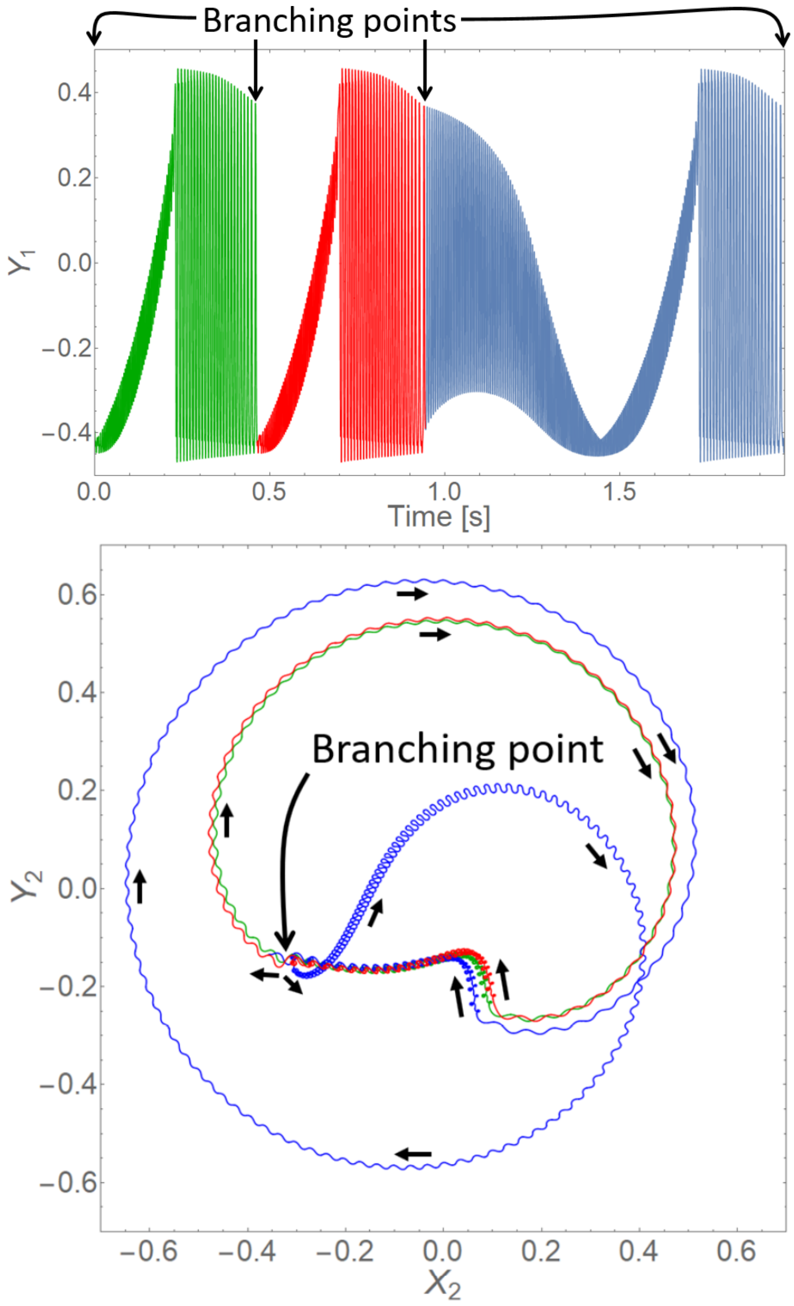

Supplementary Figure S1: Numerical simulation of the full dynamical system, Eqs. (S7)-(S10) in the RWA. Upper panel: Time evolution of one quadrature (Y_1) of the fast mode dynamics with indicated branching points. Lower panel: Corresponding trajectory in the slow variable quadrature plane (X_2, Y_2). Arrows show the evolution direction and the branching point of the RRP is indicated.

responsible for the sharp transitions in the fast variable phase space, since saddle-node bifurcations can both annihilate the stable states as well as oscillatory states. However, the observed RRPs are *not* SN-SNIC bursters and the SN-bifurcations are *not* responsible for the sharp transitions in the RRPs. Furthermore, no limit cycles are present in the fast variable plane, only stationary points. Hence, the fast variables are not tracking a stable limit cycle in its oscillatory state, which is the case in bursting dynamics. Instead, the fast variables are constantly dissipating towards one of the stable states of the fast variable plane. Despite this fact, the fast variables keep oscillating throughout the RRPs. Hence, another important feature in these RRPs is the low dissipation.

Supplementary Section 1.4 (Quasi) conservative fast dynamics

The last step to reveal the mechanism responsible for the sharp transitions in the RRPs is the fact that the effects of dissipation are very slow with respect to the fast timescale. Hence, the dynamics of the fast variables can be viewed (for a not too long period of time) as a conservative system. The corresponding Hamiltonian is

$$H = (X_1^2+Y_1^2)/2-\sqrt{3}(X_1^2+Y_1^2)^2/4-\epsilon g_{rs}(X_1^3X_2-Y_1^3Y_2+3Y_1Y_2X_1^2-3X_1X_2Y_1^2), \quad \text{(S13)}$$

with $\dot{X}_1 = \partial H/\partial Y_1$ and $\dot{Y}_1 = -\partial H/\partial X_1$. This is the (approximate) conservative system discussed in the main paper. Hence, the oscillations in the fast variable plane are not sustained by a stable limit cycle. They are prevented from fully relaxing to the stationary state due to the low dissipation. As a consequence of the conservative approximation, the fast variable phase space is split up into different regions by the homoclinic loops (trajectories connecting a saddle point to itself) associated with the saddle points; see colored regions in (Supplementary Fig. S2c). As long as the slow/fast-conservative picture holds, the nature of the fast dynamics in local time only depends on the geometry of the region in which the fast variables oscillate.

Supplementary Section 1.5 Period of oscillation for the conservative fast dynamics

The conservative system $\dot{X}_{1,2} = \partial H/\partial Y_{1,2}$ and $\dot{Y}_{1,2} = -\partial H/\partial X_{1,2}$, has two conserved quantities. These quantities are the Hamiltonian of the system H, which is given by Eq. (S13), and the Manley-Rowe invariant, which is given by $M = X_1^2+Y_1^2+3X_2^2+3Y_2^2$. With H and M, we can reduce the dynamical system to obtain the following single evolution equation for the square amplitude of mode 1 ($I \equiv X_1^2+Y_1^2$)

$$\dot{I}^2 = \frac{\epsilon g_{rs}}{3}I^3(M-I) - \left(H - \frac{I}{2} + \frac{\sqrt{3}I^2}{4}\right)^2. \quad \text{(S14)}$$

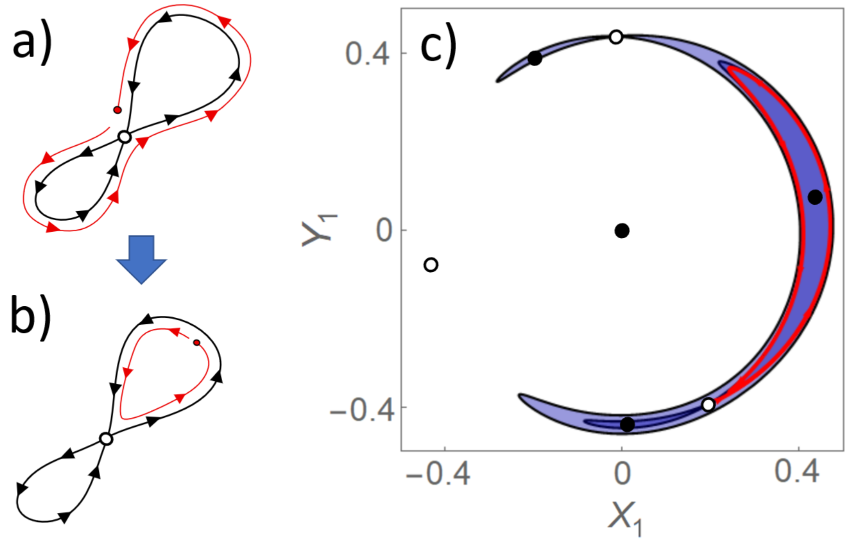

Supplementary Figure S2: Snapshot of the fast variable plane immediately after a branching event. Saddle point (hollow), stable point (black), homoclinic loops of the conservative subsystem connecting saddle points (black), trajectory of the fast variable (red). a) Immediately before the branching event, the trajectory in the fast variable plane encircles both loops around a saddle structure. b) During the branching event, the trajectory transitions into the upper loop. c) Numerical simulation immediately after the branching event. The fast system has seven fixed points at this moment, including three saddle points. The saddle point on the bottom right corresponds to the saddle point in panels (a) and (b). The red trajectory here corresponds to the red trajectory in panel (b) and corresponds to one oscillation of the full dynamics Eqs. (S7)-(S10) in the (X_1, Y_1) variable plane. The fixed points are calculated from the Hamiltonian system Eq. (S13) where the values of the variables (X_2, Y_2) are fixed (treated as constants) and equal to whatever values they have according to the full dynamics at the corresponding moment in time. Qualitatively different regions (different shadings) are separated by the homoclinic loops (black) connecting the saddle points. The branching of RRPs occur since the slow-timescale dynamics slowly deforms the connected loops, which occasionally pushes the state of the system (red) into another region. Note that the trajectories connected to the leftmost saddle point are not drawn for enhanced visibility.

From Eq. (S14), we find that the period of the fast dynamics T is the solution of the following elliptic integral [6]

$$T = 2\int_{I_1}^{I_2} dI \left[\frac{\epsilon g_{rs}}{3} I^3 (M - I) - \left(H - \frac{I}{2} + \frac{\sqrt{3}I^2}{4}\right)^2\right]^{-1/2}, \tag{S15}$$

where I_1 and I_2 are the turning points at which $\dot{I}$ in Eq. (S14) equal to zero.

Although the global structure of the response is determined with respect to the dissipation rates γ_1 and γ_2 as well as the drive strength f and detuning Δ_{IR}, the dynamics in the 2D fast-variable plane at a point along an RDS is well approximated by the conservative description and the period of the intermediate oscillations is therefore well approximated by Eq. (S15).

Supplementary Section 1.6 Branching points - breakdown of the approximations

The slow/fast-conservative picture only holds as long as conditions 1 and 2 in Supplementary Section 1.2 are fulfilled. One complication is that the conservative snapshot of the fast-variable plane is being continually updated, since the variables (X_2, Y_2) slowly evolve, and the (nearly) conservative loops evolve with them. One consequence of this is that the number of fixed points in the fast variable plane change, due to SN-bifurcations induced by the changing bifurcation parameters (X_2, Y_2). More importantly, another consequence is that the fast oscillating variables occasionally approach one of the saddle points. When this happens, condition 2 in Supplementary Section 1.2 breaks down, since the right-hand side of Eqs. (S7)-(S8) becomes small. As a consequence, the fast dynamics slows down, breaking the separation of timescales. Note that the approached saddle structure is only a saddle point in the fast-variable plane. In fact, in the full four-dimensional dynamics, it is not a saddle point, but a higher-dimensional saddle type structure. When the dynamics encounters the saddle structure, the full dynamics expels the state away from the saddle structure in one of its repelling directions, whereafter the slow/fast-conservative picture holds once again. During the transition, the slow variables have not had time to move any significant amount. Hence, the conservative snapshot after and before the branching event look almost identical. But, the fast variables have moved into or been ejected out from one of the conservative homoclinic loops. Hence, even though these homoclinic loops are only virtual approximate constructions, they are excellent indications of how the fast-variable dynamics will transform during the branching event.

This view also provides a simple picture of the branching mechanism; during the branching event, the fast dynamics make a sharp transition from encircling two homoclinic loops to suddenly performing oscillations within one of the two loops. Hence, the feedback (of the average fast variable position) into the slow dynamics can be very different, depending on which of the two loops have captured the fast variable state (similar

to resonant capture [7]). As a consequence, the dynamics can branch off into one of two different directions, with distinct long-term dynamics; see the branching point in Supplementary Fig. S1 lower panel and the accompanying movie to this supplementary information.

Description of the movie: Experimental results of the fast variable plane are shown in the left panel. Numerical simulation corresponding to Supplementary Fig. S2, for each instance during the entire RRP, are shown in the middle panel. Time evolution of fast variable Y_1 is shown in the right panel. To more clearly visualize the qualitative agreement between the experimentally measured and numerically simulated results, the time series of the measured trajectory has been piecewise stretched and compressed so that its dynamical features align with the same features in the simulated response.

Supplementary Section 1.7 Robust dynamic structures and robust response patterns

The dynamics in this article are trajectories on a so called "strange attractor," i.e., a subset of the phase space which attracts all nearby trajectories and where the dynamics of two nearby trajectories diverge over time [1]. However, the dynamics presented in this work fulfills additional conditions which defines a subclass or family of strange attractors. We refer to this family as robust response patterns (RRPs), which is defined by the following set of characteristics. The main idea is that the strange attractor can be split into a set of discrete robust dynamic structures (RDS). The RDSs are connected by branching mechanisms, which are the origin of the divergence of nearby trajectories. The strange attractor is composed of a stochastic sequence of RDSs.

The RDSs are similar in spirit to the large amplitude oscillations (LAO) and small amplitude oscillations (SAO) that constitute a typical mixed-mode oscillation (MMO) [2]. In both MMOs and RRPs the components, namely RDSs and L/SAOs, are easily identified but not easily described in formal mathematical terms. Therefore, just as in MMOs [2], our description focuses on the qualitative nature of these structures and how they are connected.

There are two primary distinctions between our RRPs and typical MMOs. First, each RDS is composed of both LAO and SAO, so the decompositions of the attractors are different. Second, in many MMOs the branching points correspond to saddle-node bifurcations in the reduced slow time system (more about this below). In contrast, in the present system, the branching points correspond to events where a slowly evolving oscillatory behavior of the fast time system encounters a saddle point, resulting in its transition to another RDS. In contrast to typical MMO dynamics which exhibits transitions from one L/SAO to one L/SAO, a key distinction of RRPs is that the branching mechanism in RRPs involve switching from one incoming to any of **two or more** outgoing RDSs. This opens up the possibility to control the execution of the RRPs. Also, in the present study we do not investigate in detail periodic versions of RRPs nor how the system changes

with parameters; these subjects are left for future work.

i) The RDSs are discrete. For example, at the level of bias presented in Fig.S1, the full strange attractor is effectively described by only the red and blue paths, although small deviations of the two exists. In this example, the green path is a small deviation of the red path but well separated from the blue. All following paths will be small deviations of the red and blue paths. That is, the space that the strange attractor occupies is encapsulated within the network of RDSs which can be viewed as "tubes" that begin and end near branching points. In this way the RDSs work as "links" between the saddle structures that represent the branching points. In contrast, the space outside the RDSs are in practice never visited by the dynamics after it settles onto the attractor. Note that two RDSs do not have to link the same branching points. For example, the strange attractor in Fig. S1 only involves one branching point and two RDSs, whereas the strange attractor in Fig.4d of the main paper involves two branching points and three RDSs.

Interestingly, most of the time, the trajectory flows along an RDS where the finite-time-local-maximal Lyapounov exponent is ~ 0. However, close to the branching points the finite-time-local-maximal Lyapounov exponent is considerably positive and fast divergence occurs for nearby paths. Hence, the global (infinite time) maximal Lyapounov exponent of the strange attractor will be positive due to the switching events at the branching points.

ii) The RDSs are robust to noise. Small disturbances (noise or control pulses) that push a trajectory away from an RDS are pulled back towards the RDS when the system is sufficiently removed from branching points. Such disturbances simply alter the specific trajectory that will be followed within the RDS. An illustrative example can be seen in Fig.S1, where the dynamics has been simulated for a time $t \gg 1/\gamma$ and the green path is ϵ-close to the red path but does not asymptotically approach the red path.

iii) The RDSs exhibit slow-fast dynamics. There is a clear separation of timescales

$$\dot{\vec{x}} = \vec{f}(\vec{x}, \vec{y}), \tag{S16}$$

$$\dot{\vec{y}} = \epsilon\ \vec{h}(\vec{x}, \vec{y}), \tag{S17}$$

where $\epsilon \ll 1$ separates the fast-variable subsystem $\vec{x}$ from the slow-variable subsystem $\vec{y}$ and $\vec{g}$ and $\vec{h}$ are nonlinear functions.

Furthermore, the RDSs exhibit oscillations in the fast-variable plane. Hence, the RDSs require at least two fast variables which enable the oscillation and at least one slow variable, i.e.,

$$\dim(\vec{x}) \geq 2, \tag{S18}$$

$$\dim(\vec{y}) \geq 1. \tag{S19}$$

Note, for the dynamics presented in this work, the fast oscillatory loops are not limit cycles but slowly evolving trajectories. However, the trajectory never reaches a steady state since the dissipation is as slow as the dynamics of the slow variables. An effective

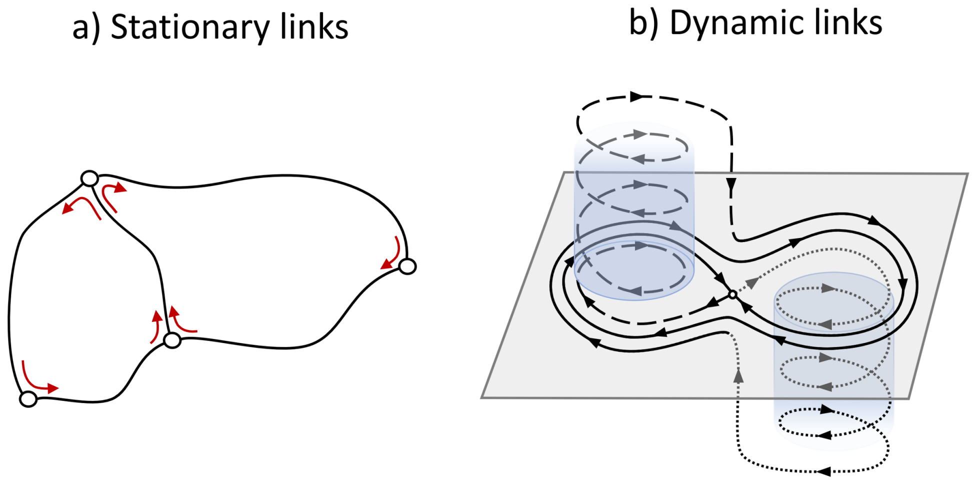

Supplementary Figure S3: Simplified illustration of RDS dynamics without and with oscillations. a) Without oscillations a trajectory relaxes towards a "stationary point" (in the fast-variable plane). This stationary point evolves on the slow timescale and the trajectory tracks it. b) With oscillations, the fast variable dynamics is more complicated, which is the case in RDSs. In this case, the RDSs can be viewed as tubes where the flow spirals along the tube walls, and a family of such spirals exists within the tube.

description of the RDSs is to view them as tubes which connect the branching points, inside which the flow is spiraling (see Fig. S3).

iv) Branching points. A *branching point* is a point where the trajectory comes sufficiently close to one of the saddle structures (corresponding to a saddle equilibrium in the reduced fast-variable plane) and the trajectory is ejected along the unstable manifold. The trajectory on the unstable manifold dictates the RDS to be subsequently followed. Note that this type of transition is induced by the drift in the slow-variable plane and cannot occur in a system with only a 2D-phase space. Also, note that the sensitivity to small disturbances at these points is what allows the type of pulse control utilized in the present work.

v) Robust response patterns (RRPs) are trajectories along the network of RDSs. The RDSs constitute a network of discrete links in phase space, which are connected (and switched between) at the branching points. The time evolution of the dynamics flows along a strange attractor constituted by repetitions of the RDSs. Depending on the level of bias, the harmonic driving signal, and/or control inputs, different robust response patterns can be achieved. The RRPs consist of high-dimensional saddle structures, which include the branching points that are linked by RDSs. Note that the saddle structures are not stationary points of the global system.

Supplementary Section 1.8 Conditions for RDSs in 3:1 internal resonance

In this article, a concrete example of the RDS phenomenon is demonstrated in a 3:1 internal resonance between two mechanical modes. Here, the separations of timescales are given by

$$1 \gg g/\alpha_1 \gg \gamma_2/|\Omega - \omega_1| >\sim \gamma_1/|\Omega - \omega_1|, \tag{S20}$$

i.e., the dissipation is the slowest timescale, and the phenomenon occurs close to the 3:1 internal resonance

$$|\omega_1 - \omega_2/3| \sim< \gamma_2. \tag{S21}$$

The system also needs to be operated at the high amplitude response branch of the Duffing curve of mode 1, i.e., requiring strong enough drive strength.

Supplementary Section 1.9 Stationary biasing of the branching mechanism

The RRP can be controlled in two ways, i) with small control pulses (see section Supplementary Section 2), and ii) by larger stationary biasing of the drive parameters. By stationary adjusting of the drive parameters, the response can be biased into executing only one or a mix of response patterns, as seen in (Supplementary Fig. S4). Hence, by tuning these parameters the dynamics can be tuned into and away from the saddle structure and the branching mechanisms can thereby be turned on and off. The simulation of the noise free model shows that the system can be biased into exclusively repeating either the red and blue structure in (Supplementary Fig. S1 top). In the measurements, we see up to 3 seconds of uninterrupted execution of the red structure (Fig. 1f in main paper). We deem that the interruptions are likely due to noise in the physical device. Inherent noise in the system blurs the distinct regions over which a specific structure is executed. We have determined from the numerical and experimental results that the parameter values allow for execution of primarily a single structure to occur even with noise.

Supplementary Section 2 Experimental implementation of control pulses

The resonator is a microelectromechanical structure (MEMS) consisting of three, 3 μm wide, 10 μm thick, and 500 μm long, doubly clamped, single crystal silicon beams connected at their center to two, 200 μm long, comb drives (25 combs) used for forcing and sensing. A schematic of the MEMS can be found in Fig. 1a of the main text. Details

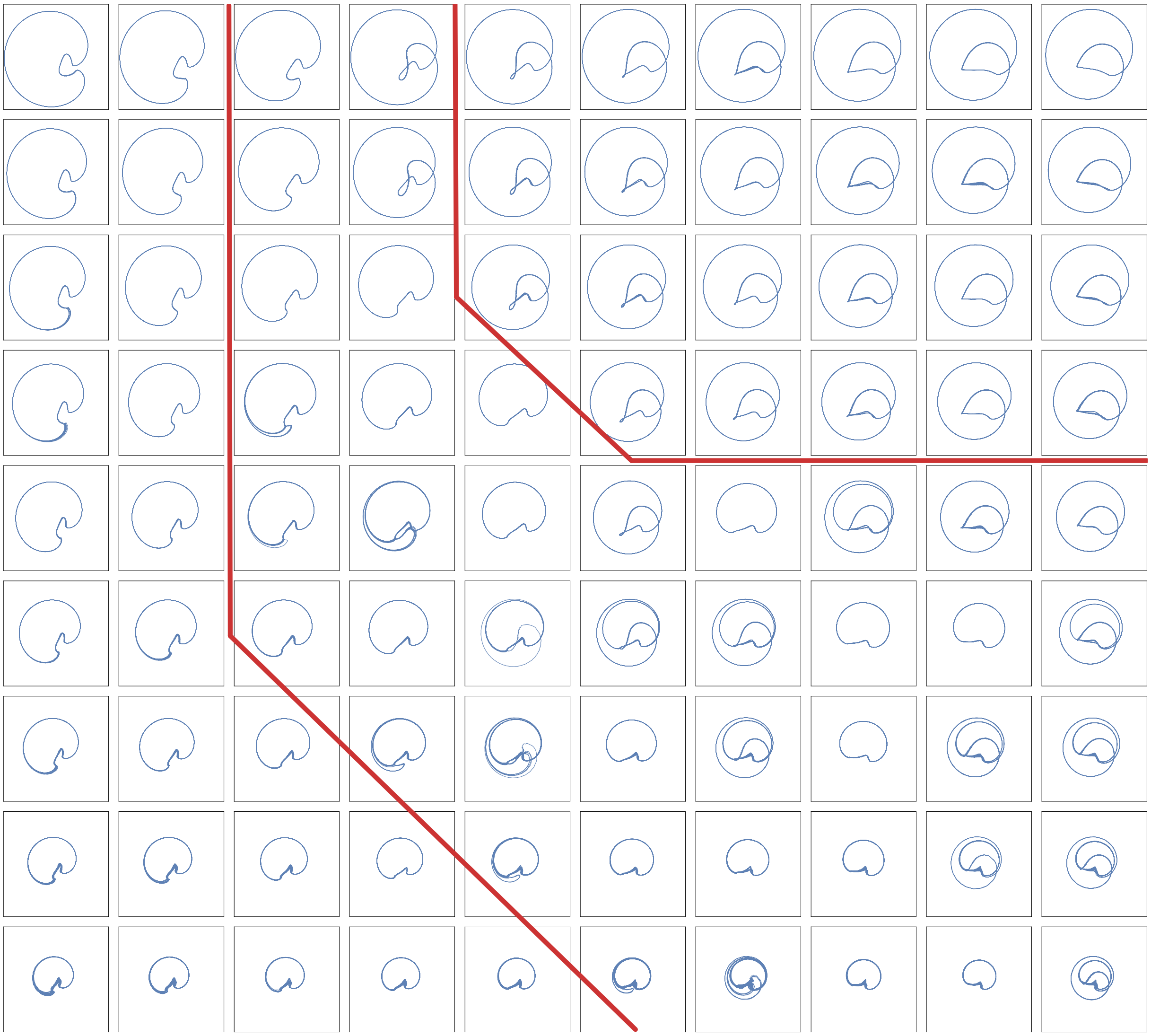

Supplementary Figure S4: Simulation of the response for different drive parameters (bottom to top): scaled drive amplitude f_{rs} = 0.0015 to 0.0055 in steps of 0.0005, (left to right): drive frequency Δ_{IRes} = -0.4 Hz to 1.4 Hz in steps of 0.2 Hz. Red lines are guides for the eye to see the separated regions exhibiting different response structures.

about the experimental measurements of the MEMS response can be found in previous work [4, 5, 8]. As a brief summary of these experiments, the MEMS resonator is driven by a sinusoidal signal from a Zurich Instruments UHFLI lock-in amplifier (212 mVrms). The body of the MEMS is connected to a bias tee with a 6.4 V DC potential and a 1 Vrms, 1 MHz, constant frequency, AC signal. The response signal from the MEMS is sent through a transimpedance amplifier and is recorded by the same lock-in amplifier. The signal is demodulated at the frequency difference between the 1 MHz signal and the drive frequency. The natural frequencies and dissipation rates of the two modes are determined from open-loop measurements using small driving forces. The Duffing nonlinearity parameter is determined by fitting the amplitude-frequency response to the driving amplitudes, for amplitudes in the nonlinear regime but below those of the IR. The coupling coefficient is determined by fitting the experimental data of the bifurcation points near the internal resonance regime, (Fig. 1c) in the main paper (for details about the fitting parameters, see [4]).

The forcing parameters for the resonator are set to a region of internal resonance where the flexural mode and the torsional mode interact. For a drive voltage of 212 mVrms, the drive frequency is increased through the internal resonance regime with responses seen in (Fig. 1d-f) of the main paper. To control the response of the resonator at the branching points, an arbitrary waveform generator function in the lock-in amplifier is used. The output from the arbitrary waveform generator is triggered by amplitude set points of the measured signal coming from the MEMS device. Short voltage pulses are added to the sinusoidal output from the lock-in, i.e. the amplitude of the ≈64 kHz drive signal is slightly increased for a duration of 10 ms.

To determine when to apply the stimulus pulses, we measure the period of the fast variable Y_1 as the resonator encircles the outer loop, see (Fig. 3b) in the main paper. When the resonator approaches the saddle structure, the period of these oscillations becomes longer (Supplementary Fig. S5), which is true for the generic case when the dynamics approach a saddle point or saddle-structure of any type, due to the slow behavior nearby [9]. Hence, an indication that the fast variable dynamics is about to hit the saddle structure is therefore that the period of the fast variable oscillations exceed the experimentally fitted threshold of 9.3 ms (measured each time Y_1 passes zero from below). To control the outcome of the branching event, we apply timed pulses. We found that the timing was the most critical component of the control pulses. The additional amplitude needed to control the outcomes was estimated in simulations to be 20% of the total amplitude, but the exact values used were fine tuned experimentally. To execute the upper loop response as shown in Fig. 3d-f in the main paper, we i) observe the period exceed the threshold, ii) wait 7 ms, iii) apply a 14 mVrms pulse with a duration of 10 ms (Supplementary Fig. S6a). Conversely, in order to execute the lower loop response as shown in (Fig. 3g-i) in the main paper, we i) observe the period exceed the threshold, ii) wait 2 ms, iii) apply a 35 mVrms pulse with a duration of 10 ms (Supplementary Fig. S6b). The applied pulses change the trajectory of the resonator as it approaches the saddle point and cause the dynamic state

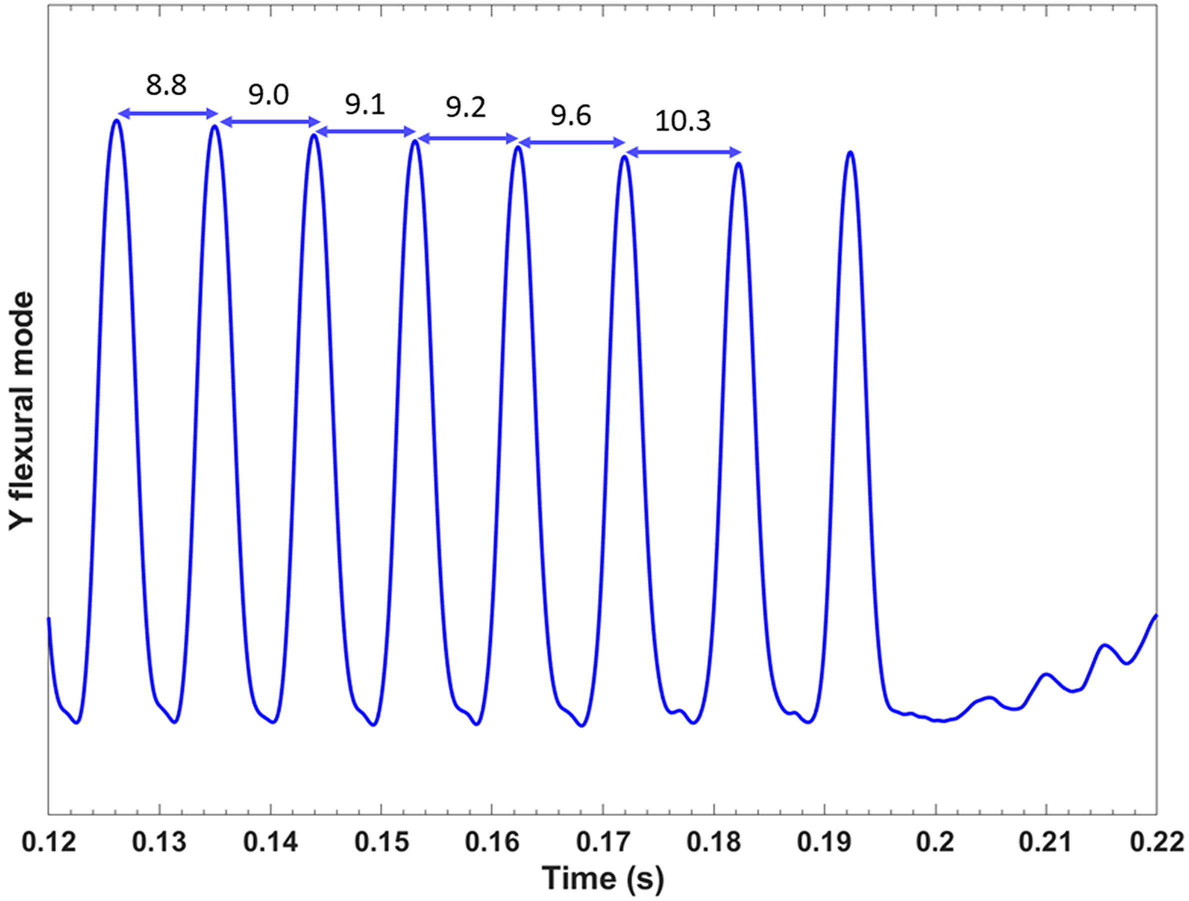

Supplementary Figure S5: Experimentally measured response of the resonator as the resonator approaches the saddle structure, showing the increasing period between oscillations as the dynamics approaches the saddle structure at approximately 0.2s. The values for the periods are in ms.

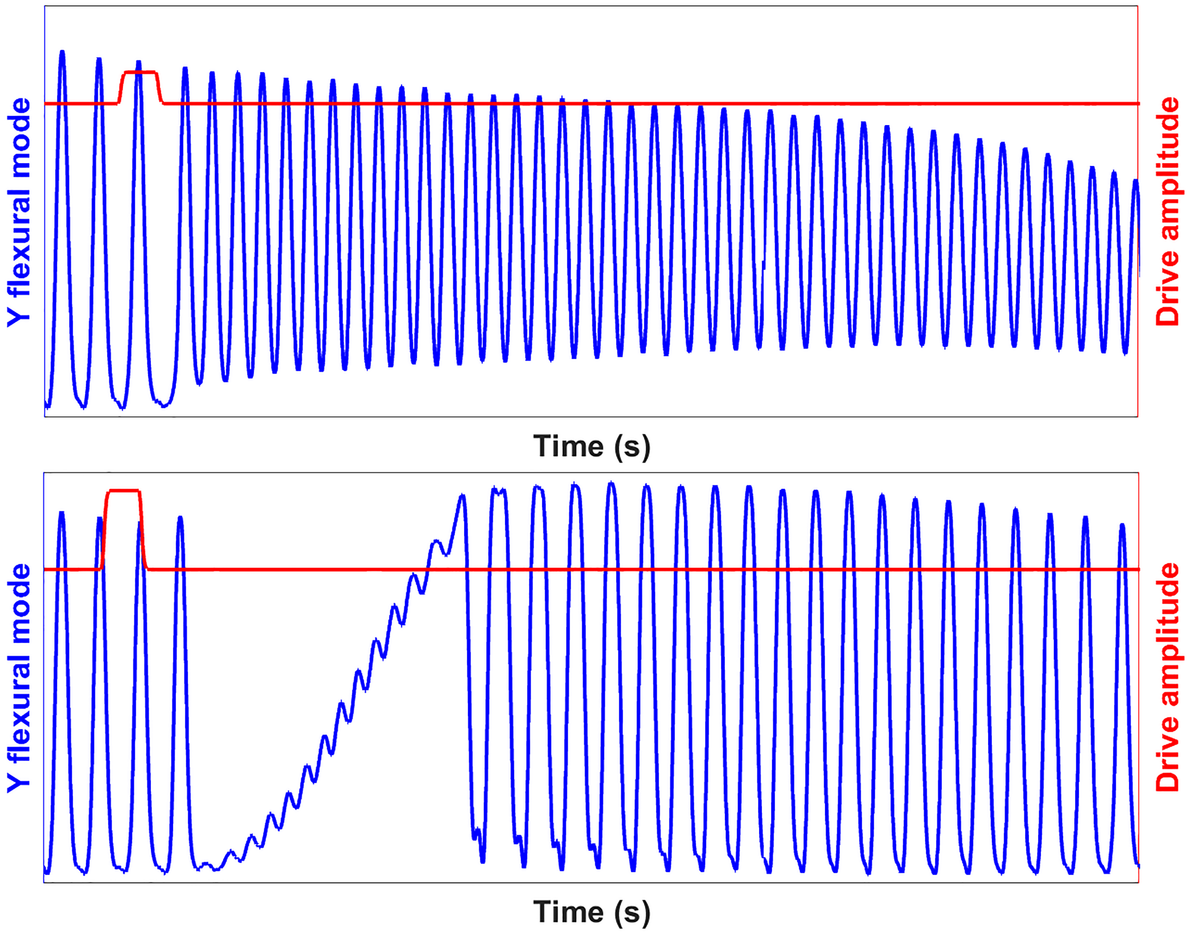

Supplementary Figure S6: Experimentally measured response of the resonator to two pulses with different amplitudes and timing. a) Resonator response to a 10 ms pulse duration with an amplitude of 14 mVrms. The timing and duration of the stimulus causes the resonator to execute the upper loop response, see (Fig. 3d) in the main paper. b) Resonator response to a 10 ms pulse with amplitude 35 mVrms. The timing and duration of the stimulus causes the resonator to execute the lower loop response, see (Fig. 3g) in the main paper].

to be trapped into one of the two loops, which branches the behavior into different robust RRPs.

Supplementary References

[1] Guckenheimer, J. & Holmes, P. *Nonlinear oscillations, dynamical systems, and bifurcations of vector fields*, vol. 42 (Springer Science & Business Media, 2013).

[2] Desroches, M. *et al.* Mixed-mode oscillations with multiple time scales. *Siam Review* **54**, 211–288 (2012).

[3] Izhikevich, E. M. *Dynamical systems in neuroscience* (MIT press, 2007).

[4] Czaplewski, D. A., Strachan, S., Shoshani, O., Shaw, S. W. & López, D. Bifurcation diagram and dynamic response of a mems resonator with a 1: 3 internal resonance. *Applied Physics Letters* **114**, 254104 (2019).

[5] Czaplewski, D. A. *et al.* Bifurcation generated mechanical frequency comb. *Physical review letters* **121**, 244302 (2018).

[6] Shoshani, O., Shaw, S. W. & Dykman, M. I. Anomalous decay of nanomechanical modes going through nonlinear resonance. *Scientific Reports* **7**, 18091 (2017). URL `https://doi.org/10.1038/s41598-017-17184-6`.

[7] Rand, R., Kinsey., R. & Mingori, D. Dynamics of spinup through resonance. *International Journal of Non-Linear Mechanics* **27**, 489 – 502 (1992).

[8] Antonio, D., Zanette, D. H. & López, D. Frequency stabilization in nonlinear micromechanical oscillators. *Nature communications* **3**, 1–6 (2012).

[9] Strogatz, S. H. *Nonlinear dynamics and chaos: with applications to physics, biology, chemistry, and engineering* (CRC press, 2018).
