# Supplementary material for: Controllable branching of robust response patterns in nonlinear mechanical resonators
Source: Nat Commun. 2023 Jan 11;14:161. doi: 10.1038/s41467-022-35685-5 (PMC9834403; doi:10.1038/s41467-022-35685-5)
Supplement: Supplementary file 3 — Description of Additional Supplementary Files [file 41467_2022_35685_MOESM3_ESM.pdf]

Descriptions of Additional Supplementary Files

Supplementary Movie: Experimental results of the fast variable plane are shown in the left panel. Numerical simulation for each instance during the entire RRP, are shown in the middle panel. Time evolution of the fast variable, Y_1, is shown in the right panel. To more clearly visualize the qualitative agreement between the experimentally measured and numerically simulated results, the time series of the measured trajectory has been piecewise stretched and compressed so that its dynamical features align with the same features in the simulated response.
